# Evaluating an implementation model of evidence-based therapy for eating disorders in non-specialist regional mental health settings

**DOI:** 10.1186/s40337-022-00695-7

**Published:** 2022-11-17

**Authors:** Catherine Johnson, Lesley Cook, Kath Cadman, Thu Andersen, Paul Williamson, Tracey D. Wade

**Affiliations:** 1 Blackbird Initiative, Flinders University, Adelaide, SA Australia; 2 Partners in Practice, Sydney, Australia; 3 The Butterfly Foundation, Crows Nest, Australia

**Keywords:** Community mental health, Implementation, Dissemination, Evidence-based, In-session weighing, Case conferencing, Dietetics, Research-practice gap

## Abstract

**Background:**

Many people with eating disorders (EDs) either do not access treatment, access it well after symptoms first start, or drop out of treatment. This study evaluated ways to improve early access to evidence-based interventions for those with EDs in a non-specialist community setting.

**Methods:**

In an Australian regional community, links were formed between general medical practitioners and treatment providers (psychologists, mental health social workers and dietitians), who received ongoing training, feedback and support. Service users had access to 20–40 subsidised treatment sessions. Data were collected from 143 patients over 18 months. Our outcomes are reported according to the RE-AIM implementation framework: Reach (we measured uptake and treatment completion); Effectiveness (impact on disordered eating cognitions, body mass index, remission, and moderators of effectiveness including illness duration, previous treatment, presence of comorbidities, presence of a normative level of disordered eating, presence of any ED behaviours, weighing in treatment, multidisciplinary case conferencing, number of dietetic sessions); Adoption (drop-out and predictors); Implementation (barriers encountered); Maintenance (subsequent activity designed to embed new practices).

**Results:**

Treatment was completed by 71%; significant large decreases in eating disorder cognitions were achieved; remission was obtained by 37% (intent-to-treat). Treatment completion was predicted by lower baseline levels of disordered eating, uptake of ≥ 3 dietetic sessions, and ≥ 2 team case conferences. Greater improvement over time was predicted by regular case conferencing and in-session weighing.

**Conclusions:**

Implementation of this model in a regional community setting produced completion rates and outcomes comparable to those found in specialist clinical trials of ED treatments. Service providers identified care coordination as the most important factor to connect users to services and help navigate barriers to ongoing treatment.

*Trial Registration:* This research was an invited evaluation of a project implemented by the Australian Department of Health. The project did not introduce any new clinical practice but sought to improve access to evidence-based multidisciplinary treatment for people with EDs by removing four known systemic barriers: securing an accurate diagnosis, availability of multidisciplinary treatment, cost of treatment, and intensity of treatment. As such, the project did not require trial registration. Notwithstanding, this evaluation obtained ethics approval (Bellberry Human Research Ethics Committee, Application No: 2018-09-728-FR-1).

**Supplementary Information:**

The online version contains supplementary material available at 10.1186/s40337-022-00695-7.

## Plain English summary

Many people with eating disorders (EDs) either do not access treatment, access it well after symptoms first start, or drop out of treatment. This study evaluated ways to improve early access to the best treatments for those with EDs in regional Australia. Links were formed between general medical practitioners and treatment providers (such as psychologists and dietitians) who received ongoing training, feedback and support. This approach achieved completion rates and outcomes equivalent to those found in specialised clinical trials of ED treatments. A key finding was the benefit of a care coordinator to connect users to services and help navigate barriers to ongoing treatment.

**Supplementary Information:**

The online version contains supplementary material available at 10.1186/s40337-022-00695-7.

## Background

Dissemination of evidence-based interventions (EBI) from controlled research environments to routine clinical care in community settings is widely recognised as problematic in terms of ensuring that consumers receive effective treatments [1]. This has been conceptualised as a research practice gap (what works does not get translated into ongoing clinical practice) and a treatment gap (people do not have access to treatment). Models to address the research practice gap target improved and accessible practitioner training, for example scalable web-based methods [2] with outcomes including therapist competence and adherence. Models addressing the treatment gap include approaches of "best buy" therapies (selected based on cost-effectiveness, and feasibility for the setting; e.g., [3]) and higher-level support and policy such as training more but less specialised practitioners (e.g., [4]). In these models, the outcome of most interest is the reach of the treatment and its outcome.

Eating disorders (EDs) provide a complex example of both the research and treatment gaps in mental health care. EDs are associated with suboptimal uptake of evidence-based practice in community settings, together with low and late treatment uptake and high dropout rates. Factors known to impede access to evidence-based therapy for EDs include the high costs of treatment due to the multidisciplinary character and long duration of treatment [5] together with low levels of knowledge of EDs amongst clinicians [6] and lack of confidence to treat [7, 8]. An Australian study found that 73% of clinicians in a regional health service had little confidence to work with clients with EDs, with a strong correlation between training and confidence to treat [9].

These issues have been thrown into sharp relief in Australia by the recent expansion of the federal government funded Medical Benefits Schedule (MBS) which subsidises evidence-based treatment for severe EDs that meet DSM-5 [10] diagnostic criteria [11]. The federal government also funded delivery and evaluation of a model to increase availability of EBIs for people with all EDs (not just severe presentations) through community mental health settings in regional Australia. This project (reported here) addressed two key issues related to the research practice and treatment gaps, practitioner knowledge and skills to treat, together with the cost of treatment. To administer this project within a limited time frame, the project also introduced a care coordination role with responsibility for helping clinicians to implement team care whilst also helping patients to navigate the care system and stay engaged in treatment. The model created pathways to treatment that included payment for sessions (a mixture of MBS and patient payment), variable appointment availability (both in terms of time available and geographic location), and variable level of therapist qualifications and expertise in ED therapy.

To organise reporting of the outcomes of the model, we adopt the Reach, Effectiveness, Adoption, Implementation and Maintenance (RE-AIM) framework [12]. We examine the success of the model using the following outcomes. First, in terms of **Reach**, we report on the number of patient referrals and uptake rates and treatment completion. Second, we report on individual **Effectiveness**, in terms of disordered eating, body mass index (BMI) and remission. We examine moderators (fixed baseline and practice adherence) of effectiveness of patient dropout and outcome. Third, we report on **Adoption**, in terms of the characteristics of the client group who engaged in treatment, and predictors of patient dropout and outcome (fixed baseline moderators). Fourth, we report on problems encountered in Therapist Implementation and strategies used to manage these i.e., barriers and facilitators. Finally, we examine strategies in place to **Maintain** the sustainability of the implementation.

## Methods

### The model

The Sunshine Coast ED Access Trial was an initiative of the Australian Department of Health who provided funding to the Butterfly Foundation in partnership with a Primary Health Network (PHN) in regional Queensland to improve access to ED treatment. The aim was to investigate what effect introduction of expanded MBS items would have, and hence the model was embedded in the context of the MBS (https://www.anzaed.org.au/newmbsitems/) which specifies: that referral and review be provided by a medical practitioner; the inclusion of severe EDs only; the approved treatment modalities; and the number of treatment sessions provided. MBS items were not only available for psychological therapy but also sessions with a dietitian.

The Sunshine Coast is a periurban region of 1633 square kilometres with mixed urban and rural characteristics. The population of 346,648 is clustered in small communities, primarily along the coastal fringe of the region. The coastal area has a strong economy, particularly in health care, education, and tourism and an unemployment rate comparable to the national average at 4% in March 2022. The more rural hinterland has pockets of relative poverty with a higher rate of unemployment at 6.3% in March 2022. Public transport in the region is limited and the need for reliable transport is identified as a critical development issue.

The data examined in the current report were collected between February 2019 and 18 August 2021, a period of 30 months. The research was approved by the Bellberry Human Research Ethics Committee, Application No: 2018-09-728-FR-1. From November 2019, when MBS became available for patients with severe presentations of ED, the model focused on ED not meeting the MBS criteria, including earlier intervention for people and who were seeking treatment for their ED for the first time or were seeking treatment to manage the risk of relapse after previous treatment. This latter group tended to have lower levels of symptoms. We therefore refer throughout this report to two groups: one with clinically significant symptoms (baseline mean ED-15 cognitive scores ≥ 3.38; more than 1 SD above UK norms [13]), and/or at least one ED behaviour endorsed on the ED-15) and one with normative symptoms who did not meet this threshold.

Care coordination was a critical and unique aspect of the model, supporting the development of a cohort of trained and networked service providers as well as assisting patients to navigate the care system. This required proactive engagement of general practitioners who wished to refer people with EDs for psychological assessment and treatment, and service providers who had skills in delivery of ED assessment and treatment. A Care Coordinator with a nursing background was employed to liaise between the two groups, as well as to work with service providers to triage referrals, support the formation of treatment teams, match services to patient needs, and support practical problem solving as difficulties arose. The role also maintained contact with patients and was available to help resolve barriers that could lead to disengagement from treatment.

Service providers were required to undertake free training, comprising a 3-h introductory session with an emphasis on assessment and diagnosis and at least 10 h of training in a relevant specific treatment modality. Of the ten treatment modalities approved by the MBS, the model supported delivery of three: Cognitive Behaviour Therapy-Enhanced (CBT-E), Family Based Therapy, and Specialist Supportive Clinical Management. The latter two modalities became irrelevant from November 2019, when the MBS was launched for patients with severe presentations of ED, including all presentations of anorexia nervosa. This means our group with normative symptoms could have been treated with CBT-E or other EBI in which service providers had pre-existing expertise, including guided self-help CBT, Dialectical Behaviour Therapy, or Interpersonal Psychotherapy. Trainers from the local region and nearby metropolitan services provided the training, which was assessed for suitability in terms of the alignment of the National Eating Disorder Collaboration “National Framework for Eating Disorder Training—A guide for training providers”. Service providers were required to use key measures of patient progress which enabled ongoing professional development targeted to specific gaps in delivery of effective treatment delivered in real time.

For non-specialist clinicians, integrating a new treatment modality plus a new team-oriented way of working (which at the least involved the general practitioner and could involve a dietitian) into an already established and full schedule of work can be overwhelming. Therefore, the model also advocated four anchor points to support ongoing engagement of the clinician and the patient. Two related to increasing the likelihood of basic evidence-based practice, namely sessional weighing and progress monitoring, in this case using the ED-15 [13]. Two related to creating a supportive team environment, namely dietitian involvement and case conferencing. Service providers were paid a sessional fee when they participated in case conference meetings and the treatment team submitted a summary of team discussion as evidence that the meeting occurred.

### Participants

#### Service providers

Service providers (n = 72) included the following disciplines: Clinical Psychologists (n = 20), Registered Psychologists (n = 47), and Mental Health Social Workers (n = 5). On average, practitioners had 9.75 years of experience (*SD* 5.3; range: < 1–32) in mental health. Data were not collected on years of experience treating EDs.

#### Service users

The model relied on local health service providers to identify people with EDs, refer and/or provide treatment. Referrals were accepted from February 2019 to June 2021. Eligibility criteria for service users were (1) meeting the DSM-5 [10] diagnostic criteria for an ED, (2) residing in the health network area; (3) aged over 14 years; (4) referred by a GP or other recognised health professional; and (5) assessed as safe to receive community-based treatment by a medical professional and a mental health professional.

### Design

The study design was a case series (no comparison group), where the primary outcome for patients was a short self-report measure of ED cognitions administered on a session-by-session basis. Secondary outcomes included body mass index (BMI) at baseline and end of treatment, and remission. Diagnosis by treating mental health practitioner, uptake of appointments with a dietitian, regularity of case conferencing by the treatment team, and use of in-session weighing in treatment sessions were also recorded. Data were drawn from clinician records which were deidentified for the evaluation component.

### Measures

#### Baseline demographics

Clinician diagnosis was supplied by mental health practitioners after assessment (usually over two sessions). Age, duration of illness, presence of comorbidities, socioeconomic status and previous psychological treatment for ED were drawn from patient data records.

#### Primary outcomes: eating disorder cognitions and behaviours

We used the ED-15 [13] as our primary outcome. The first ten items from the ED-15 assess ED cognitions over the preceding week (e.g., “*Compared my body negatively with others*”), rated on a 7-point Likert scale from 0 “*not at all*” to 6 “*all the time*” [6]. The last 5 items from the ED-15 assess ED behaviours using count responses, with participants reporting the frequency of objective binge eating, vomiting, laxative use, dietary restriction and driven exercise. Clinicians were instructed to collect the ED-15 measure at each session from service users, both to discuss individual results in session and to record outcome data. The ED-15 has been validated in clinical and non-clinical samples and demonstrated acceptable concurrent and convergent validity [14]. Cronbach’s alpha for the 10-item cognitive measure in the present study was 0.91 (item-total correlations > 0.41).

#### Secondary outcomes

*BMI* Height and weight were measured by the clinician at baseline; clinicians were asked to continue weighing at each session and this was examined as an outcome for patients who were underweight (BMI < 18.5).

*Remission* Remission status was based on attaining normative ED-15 cognitive scores (mean ≤ 3.38; within 1 SD of UK norms [13]) and absence of ED-15 behaviours. Last available observation was used if participants did not complete therapy.

#### Predictors of dropout and outcome

Fixed baseline moderators included: duration of illness, previous psychological treatment for an ED, presence of comorbidities, presence of a normative level of disordered eating and presence of any ED behaviours. Practice adherence predictors, recorded in service user data records, included regular weighing in treatment, regular case conferencing (meetings between treatment team members), and number of sessions provided by the dietitian. Case conferencing was eligible for rebates and suggested monthly, but frequency was at the discretion of the treatment team.

### Statistical analyses

#### Dropout: percentage and predictors

Completion was defined as either (1) Patient and service provider(s) agreed that patient was ready for discharge from ED treatment, or (2) Patient discontinued treatment after ≥ 10 sessions with a decrease in ED reported in records. This combination represents those considered to have received a sufficient dose of treatment. Logistic regression was used to test predictors of treatment completion.

#### Sessional changes over treatment and predictors

Multilevel modelling (MLM; statistical software *R* version 4.0.3) was used to analyse change over time in sessional ED-15 scores (10 cognitive items). This approach estimates trajectories of change for all participants, and accommodates incomplete data, together with data collected at differing time points for individuals. Time was modelled as weeks from baseline assessment. The following sequence of models was used. Step 1: Null model fitted to calculate the intraclass correlation coefficient (ICC) and assess whether significant variance was present in the outcome. Step 2: Growth modelling to assess whether the average variance changed over time. Both linear and quadratic components were fitted. Step 3: Random slope modelling to test whether trajectories of change varied across individuals. Step 4: Testing whether an autoregressive error structure improved model fit. Step 5: Testing interactions to see if any of the following variables predicted the trajectories of change: weight classification (underweight = BMI < 18.5), duration of illness, previous psychological treatment for ED, presence of comorbidities (Yes/No), compliance with evidence-based practice (team-based approach—use of ≥ 3 sessions provided by a dietitian; use of ≥ 2 team case conferences; weighing at ≥ 50% of visits with mental health practitioner). Post hoc, mean ED-15 scores (10 cognitive items) were used to calculate within-group effect sizes (Cohen’s *d*) to allow comparison to clinical trials [15]. Analyses were undertaken for both treatment completers and intention-to-treat (ITT) samples.

## Results

### Reach

#### Uptake

We do not have pre-implementation data available but there was very little clinical input being offered for EDs before implementation, as is typical in a regional area of Australia. The funding was deliberately targeted at an area where there were few services being offered for people with ED. Additional file 1: Fig. S1 shows the referrals for services (n = 303) of which 215 (71%) commenced treatment with service providers in the model and a further 78 accessing alternative pathways, most commonly after the MBS launch to more specialised services.

#### Treatment completion

Drop out analyses were based on cases with complete outcome data (both BMI and ED15; *N* = 143); 103 cases (72.0%) completed treatment, 15 cases (10.5%) transferred to external pathways for mental health or ED support, and 25 cases (17.5%) dropped out. For the underweight group (BMI < 18.5), 22/31 (71%) completed treatment. For those with BMI ≥ 18.5, 81/112 (72.3%) completed treatment.

### Effectiveness

#### Sessional changes over treatment

Growth modelling showed a significant improvement in ED-15 cognitions, with rate of change slowing over time, for both ITT and completer samples. Table 1 shows the start to end of treatment within group effect size change for the cognitive items of the ED-15 for various subgroups, which were all in the upper end of moderate or large.

**Table 1:** Pre- and post-treatment scores for ED15 cognitions

Group		Whole sample		Baseline BMI < 18.5		Baseline BMI ≥ 18.5	
		Pre-M *(SD)* Post-M *(SD)*	Within-group effect size *(95% CI)*	Pre-M *(SD)* Post-M *(SD)*	Within-group effect size *(95% CI)*	Pre-M *(SD)* Post-M *(SD)*	Within-group effect size *(95% CI)*
Completers	All *N* = 103 (Underweight *N* = 22)	3.47 (1.49) 2.10 (1.51)	**0.91 (0.63,1.20)**	3.13 (1.53) 1.81 (1.49)	**0.87 (0.26,1.49)**	3.57 (1.47) 2.19 (1.51)	**0.93 (.60,1.25)**
	Clinical Subsample *N* = 50 (Underweight *N* = 9)	4.57 (0.76) 2.70 (1.52)	**1.56 (1.11,2.00)**	4.53 (0.74) 2.54 (1.89)	**1.39 (0.36,2.42)**	4.57 (0.78) 2.73 (1.45)	**1.58 (1.08,2.08)**
ITT sample	All *N* = 143 (Underweight *N* = 31)	3.53 (1.41) 2.40 (1.62)	**0.74 (0.50,0.98)**	3.45 (1.49) 2.26 (1.78)	**0.73 (0.21,1.24)**	3.55 (1.40) 2.44 (1.58)	**0.74 (0.47,1.01)**
	Clinical Subsample *N* = 76 (Underweight *N* = 14)	4.47 (0.77) 3.09 (1.61)	**1.09 (0.75,1.43)**	4.66 (0.81) 3.33 (2.01)	**0.87 (0.09,1.64)**	4.43 (0.76) 3.04 (1.52)	**1.16 (0.78,1.54)**

Significant results bolded; Within group effect size is Cohen’s *d;* *M* mean

#### BMI and remission status

For those who were underweight at baseline (BMI < 18.5), 36.4% of completers and 29.0% of the ITT sample also achieved normative BMI. Overall, between 58.0% and 72.7% (completers) and 51.8% and 64.5% (ITT) achieved remission (Additional file 1: Table S1). For the subsample with clinically significant symptoms (clinical levels of ED-15 cognitions and/or ED behaviour at baseline), between 42.0 and 45.5% (completers) and 35.7 and 41.9% (ITT) had reached remission.

#### Fixed baseline moderators

**Fig. 1:**
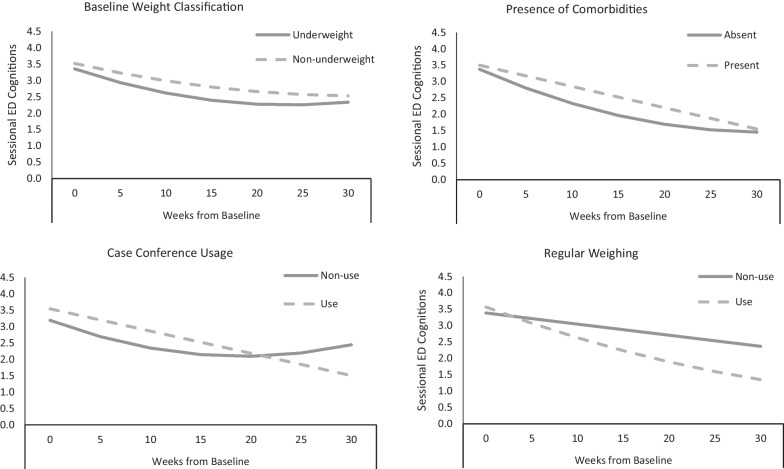
Change in ED15 cognitions over time: moderators

Predictors of rate of change over time are presented in Table 2. While change was more rapid for underweight patients, progress started to level off such that the non-underweight patients caught up with their progress by end of treatment (Fig. 1); post-hoc analyses showed a significant improvement in ED-15 cognitions scores for both underweight and non-underweight patients. For those with comorbidities, rate of improvement was slower initially compared to those without comorbidities, but the graphs suggest that the degree of improvement was similar over time.

**Table 2:** Predictors of Treatment completion and rate of improvement over time (ED15 cognitions)

Predictors		Drop out			Improvement over treatment
		ITT n = 143 (%)	Completers n = 103 (%)	Odds ratio (95% CI)	*p* value in model
Duration of illness	≥ 3 years	35.0	34.0	1.20 *(0.55, 2.65)*	.91
Previous psychological treatment for ED	Yes	29.9	26.4	0.62 *(0.29,1.35)*	.32
Weight classification	BMI < 18.5	21.0	21.7	1.07 *(0.44,2.58)*	.03*
Presence of comorbidities	Yes	89.2	88.7	0.84 *(0.26,2.79)*	.01*
Normative baseline ED15 cognitive score^a^	(≤ 3.38)	38.5	43.7	2.33 (1.03,5.26) *	NA^b^
Presence of any ED behaviours at baseline	Yes	74.1	73.8	0.94 *(0.41, 2.17)*	NA^b^
Regular weighing during treatment	(≥ 50% of visits with practitioner)	58.0	61.3	1.29 *(0.62,2.70)*	< .01*
Regular case conferencing	(≥ 2 sessions)	80.3	88.7	2.88 (1.15,7.21) *	< .01*
Regular use of Dietetics	(≥ 3 sessions)	72.0	84.9	4.92 (2.17,11.15) *	.61

*significant *p* < .05

^a^Normative split based on 1 SD above mean non-clinical scores; Tatham et al. [13]

^b^Outcome same as predictor so not tested in model

#### Practice adherence predictors

Treatment that utilised regular case conferencing was associated with an ongoing decrease in ED cognitions rather than the rebound effect seen in the group without this regular collaboration. Those patients whose clinicians weighed them regularly showed greater rates of improvement (Fig. 1). Regular case conferencing and weighing also resulted in significantly lower levels of ED-15 cognitions at end of treatment. In contrast to the trends observed for weight and comorbidity, the graphs suggest that degree of improvement remained different over time.

### Adoption

The characteristics of the client group who engaged in treatment appear in Additional file 1: Table S2. Predictors of patient dropout are shown in Table 2. Completion was predicted by normative baseline ED-15 cognitive scores, uptake of three or more sessions provided by a dietitian, and two or more treatment team case conferences.

### Implementation

Over the application of the model three barriers to implementation arose. The first was a mismatch of diagnosis between that assigned by the service provider and the profile of the patient captured in data collection. It was considered important to distinguish between anorexia nervosa and other disorders given that people with this diagnosis were meant to be referred to an alternative specialist pathway. The second was the omission of regular weighing of the patient in session. Weighing in session is a central key component of most EBI [16]. The third was the emergence of COVID.

#### Informed diagnosis

While therapists successfully identified the presence of an ED, preliminary analyses (April 2020) suggested that therapist diagnosis matched DSM-5 criteria (BMI combined with patient self-report of disordered eating behaviours) in only 37.3% of cases. It was noted anecdotally that some therapists were under-reporting symptoms so that the patient could remain eligible and that GPs generally tended to adopt a cautious approach to preliminary diagnosis by under-diagnosing. During October and November 2020, three “micro-skill” training sessions designed for busy clinicians were offered to service providers (Additional file 1: Table S3), one of which focused on diagnosis and another on weighing. At the conclusion of the evaluation (August 2021), diagnoses assigned by mental health practitioners before and after the training session were compared, noting that new patients referred post training were comparatively small. Key findings appear in Table 3. Overall, there was a lack of improvement in diagnostic accuracy, with continuance of high levels of false negative anorexia nervosa diagnoses.

**Table 3:** Impact of micro-skills training to improve diagnostic accuracy by mental health practitioners

Indicators of diagnosis accuracy	Pre-training	Post-training
Sufficient information recorded for DSM-5 diagnosis	141/167 (84.4%)	40/44 (90.9%)
Practitioner diagnosis also available	110/141 (78.0%)	36/40 (90.0%)
Practitioner diagnosis matches DSM-5 diagnosis	63/110 (57.3%)	18/36 (50.0%)
Anorexia false positive	22/141 (15.6%)	2/40 (5.0%)
Anorexia false negative	8/30 (26.7%)	4/7 (57.1%)

#### Regular weighing

Baseline data (December 2019) revealed BMI was entered in only 35.1% of patient records. To educate clinicians on the importance of weighing as part of evidence-based practice for ED, a bulletin was circulated to service providers in January 2020. This did not specify whether weighing should be open or blind. Over the subsequent four months, sessional data showed a significant increase in discussion of weight within sessions, but not in weighing frequency (Table 4). Given the concurrent increase in telehealth sessions during this period due to COVID-19 (pre-bulletin 4.0%; post-bulletin 27.9%), data for face to face sessions were also examined separately to remove this confound. Face to face sessions showed a significant increase in both discussion and weighing behaviour post bulletin, although the latter improvement was more moderate. Four months post the micro-skill training sessions, which included open collaborative weighing and non-negotiables of therapy, we repeated analysis of sessional data with results now showing a significant increase in weighing frequency in both the whole sample and the face to face subgroup. Considering the ongoing increase in telehealth sessions post COVID (19.3% in final phase of our data collection compared to 4.0% initially), we also report on weighing behaviour in this subgroup. Practitioners using telehealth could ask clients to self-weigh and self-report, or send screenshots of scale readings. Not all clients owned scales. Frequency of weight discussions and behaviour did not change from pre-bulletin to post online training within the telehealth subgroup. Compared to the face to face group, there was no significant difference across weighing practices until after micro-skills training, when weighing compliance increased in the face to face group compared to telehealth.

**Table 4:** Impact of strategies to increase sessional weighing by mental health practitioners

Time	Psychology consultations^a^ (N)	Weighed (% of sessions)		Discussed (% of sessions)	
*All sessions*					
Pre-Bulletin	475	47.6	T1 versus T2	69.9	T1 versus T2*
Post-Bulletin^b^	419	53.7	T2 versus T3	90.0	T2 versus T3
Post online Training^c^	445	60.2	T1 versus T3*	87.9	T1 versus T3*
*Face to face sessions only*					
Pre-Bulletin	446	47.8	T1 versus T2*	69.3	T1 versus T2*
Post-Bulletin^b^	281	57.3	T2 versus T3*	92.5	T2 versus T3
Post online Training^c^	351	66.7	T1 versus T3*	94.6	T1 versus T3*
*Telehealth sessions only*					
Pre-Bulletin	19	57.9	T1 versus T2	78.9	T1 versus T2
Post-Bulletin^b^	117	47.0	T2 versus T3	87.2	T2 versus T3
Post online Training^c^	86	37.2	T1 versus T3	64.0	T1 versus T3
*Between group comparisons: Face to face versus* *Telehealth*					
			T1		T1
			T2^#^		T2^#^
			T3*		T3*

*Significant

^#^Approached significance, *p* = .06–.09

^a^Excluding case conferences and assessments which often spanned two visits

^b^Four months post bulletin

^c^Four months post online training

#### COVID

The implementation was affected by COVID-19 health directives (March 2020 to June 2021). In response, access to telehealth appointments were increased, and the impact on patients was monitored via telephone contact with the Care Coordinator (n = 108). Of these 67% reported increased eating disorder symptomatology. Other difficulties reported included food security, increased symptoms of anxiety and depression, and financial difficulties. Only 30% reported reduced access to treatment. Reactions to telehealth services varied, with most respondents preferring face-to-face contact.

### Maintenance

On completion of the project, supports for the region’s eating disorder system were gradually withdrawn over a 9-month period. Participants who had not completed treatment were transferred to an alternative payment plan and continued in treatment. All of the clinicians involved with the project have continued to provide services to people with eating disorders in the region. Evaluation of this process indicated an ongoing need for access to training and care coordination to sustain the type of outcomes found during the project. More broadly, national activities that can maintain the sustainability of the implementation are now being put in place. The Federal Government have supported a partnership between the Australian and New Zealand Academy of Eating Disorders (ANZAED) and the National Eating Disorders Collaboration (NEDC) to develop a credentialing system for mental health professionals and dietitians providing treatment for people with EDs. This system commenced in 2022 and provides pathways for training and supervision. Supervision was not formally provided in the model and given its importance for changing service provider behaviour, represents an important initiative.

## Discussion

This evaluation examined a model designed to decrease the treatment gap and provide increased, early access to evidence-based treatment for people with EDs living in regional Australia. Key findings are presented according to the RE-AIM implementation framework that guided our study.

Our ability to address reach is somewhat diminished by a lack of pre-implementation data, but in an area where previously few services had been offered for ED, the uptake of services proved to be constant over the course of the study. It is possible that EDs that did not meet the severity required by the MBS criteria may not have been serviced without this model.

Across different clinical effectiveness trials targeting EDs [15] within-group Cohen’s *d* effect sizes for improvements in ED cognitions range from 1.07 to 2.37 (completers) and 0.39 to 2.37 (ITT). The effect sizes obtained in the current study for those with clinically significant symptoms ranged from 1.09 (ITT) to 1.56 (completer), commensurate with other effectiveness studies. In the whole group, where 43.7% (completers) and 38.5% (ITT) had normative baseline ED-15 scores, the improvements in ED-15 cognition scores were smaller (*d* = 0.91 and *d* = 0.74 respectively), reflecting a trial promoting early detection and intervention. Rates of remission of clinical effectiveness trials targeting EDs [15] range from 31 to 54% (end of treatment, completer samples) and 37% and 78% (end of treatment, ITT). We observed 43% (completer) and 37% (ITT) attain change that bought them to normative levels of functioning. For our ITT sample, rates of remission were at the lower end of specialist trial figures, but were mid-range for our completers. Both results are important findings using non-specialist community practitioners, but speak to the need to address dropout, considered next. As part of the examination of effectiveness, we reported on both fixed baseline moderators and practice adherence predictors of dropout and outcome, with the latter being relatively novel in the literature. Completion was predicted by normative baseline ED-15 cognitive scores, which may speak to the benefit of diagnosing and intervening early, reinforcing the recent move in the ED field to focus on the importance of early intervention [17, 18] The uptake of three or more sessions provided by a dietitian also predicted retention, perhaps reflecting the preference of consumers for referral to a dietitian for assessment, education, and guidance about nutrition, significantly higher than endorsement of such practice by ED clinicians [19]. Use of two or more case conferences predicted both retention and better outcome. An examination of case notes suggested case conferences were more likely to be held when difficulties were experienced in treatment or with patient retention. This may suggest a protective effect of multidisciplinary practice. Alternatively, it could suggest that more conscientious therapists provided better treatment. Regular in-session weighing also predicted better outcome. Regular in-session weighing is recommended as a therapeutic technique of exposure for all ED diagnoses. Being weighed did not impact on dropout, suggesting this was well tolerated by patients. Regular weighing and use of a progress measures such as ED-15 may have provided helpful anchoring practices for therapists, prompting broader fidelity to EBI.

Adoption of the treatment by patients was good. A recent systematic review reported a 24% dropout rate from treatment for EDs [20]. Dropout rates in the current study were slightly lower at 17.5%, which may reflect short waiting times (an average of 2 weeks), given increased wait-list times have been found to predict dropout from therapy for EDs [21]. Additionally, assistance from the Care Coordinator to connect patients to service providers in their area and to clinicians offering no-gap services for low income patients may have contributed to a lower dropout. Indeed, feedback from service providers in the transition phase post-project showed 100% agreement that Care Coordination was the highest priority for an effective ED system of care.

Three issues impacted on implementation. The first was inaccurate differentiation between anorexia nervosa and other EDs. Use of a training on diagnosis did not significantly improve accuracy of diagnosis, and in fact there was a slight decline in accuracy. Given that inaccurate diagnoses were mainly driven by false negative anorexia nervosa diagnoses, this inaccuracy may have been maintained by lack of eligibility for people with anorexia nervosa, given an alternative MBS pathway became available. We also noted low levels of in-session weighing. While weighing is a routine practice for EBI in EDs [16] it is the least endorsed as being received by people describing what occurred when they received cognitive behaviour therapy [6]. Use of both a bulletin and a workshop on the importance of this weighing supplemented by strategies to manage patient refusal significantly improved occurrence of in-session weighing for face to face appointments, from 48% of sessions to 66.7%. This represents a higher level than the 39% of patients who reported receiving sessional weighing when doing cognitive behaviour therapy in community settings [6]. The occurrence of COVID over the evaluation was also noted, with some decrease in access to treatment reported by patients, who also expressed reservations about telehealth. Frequency of telehealth consultations with psychologists showed an initial surge from baseline to mid-project (4.0 to 27.9%) which remained elevated as this project concluded (19.3%) and may represent a lasting change. In contrast to face to face services, neither the practitioner bulletin nor workshop impacted on weighing behaviour for telehealth services. Given the ongoing uptake of telehealth for convenience in the post-COVID era, specific education to improve compliance with evidence-based weight practices during remote consultation is recommended.The project was delivered over three years with an additional 9 months of evaluation post-project. The continued demand for training and care coordination during and post-project suggests that closing the treatment gap may require sustained support over several years. Care coordination was identified as one method of ensuring that providers are continuously supported to maintain practice standards.

### Limitations

This translational study is informative and unique, but findings should be interpreted in the context of the following limitations. First, this is a case series; real-world evaluation helps us to understand how evidence-based treatment translates in practice, but we have no comparison with previous practice which would inform the Reach component of the RE-AIM framework. Second, a model of higher-level support and policy is culturally specific but does allow for some implications to diverse settings. Third, pre-registration of the study was not possible given the dynamic nature of the research which was to respond to issues as they emerged. Fourth, even in our ITT analyses, only the data of 67% of treatment commencers were analysed due to either incomplete data or patients were still undergoing treatment. Fifth, the type of referrals differed over the lifetime of this study, with reduced referral of people with anorexia nervosa, reflecting national policy changes. Sixth, we had no measure of therapist adherence to the EBIs used. Seventh, our definition of drop-out is specific to this study, and difficult to compare to other studies, but we note that the definition of drop-out has differed widely across different treatment studies of ED, with little uniformity [20]. Finally, our outcome data, including remission, only refers to the last week. This reflects the practical difficulties posed by dissemination studies of achieving satisfactory response rates with longer but less frequent questionnaires [18], with more complete data provided by shorter sessions measures [4, 18].

### Future research directions

There is a paucity of the evaluation of models which seek to close the research and treatment gaps in settings of non-specialist community mental health care. Given that the need for ED treatment outstrips the availability of specialist care [1], equipping mental health practitioners to offer fit-for-purpose therapy to patients with EDs is a critical endeavour. The systems-change model evaluated here which was implemented in the context of existing health policies and priorities suggests that acceptable outcomes can be obtained with a higher-level support and policy. Direct comparisons of different models designed to close the gaps are required, and the inclusion of health economic evaluations of these approaches compared to specialist care would also be valuable.

## Conclusions

This study evaluated a model to increase access to evidence-based multidisciplinary treatment for people with eating disorders (EDs) in regional Australia. Associated with low, delayed uptake and high dropout rates, EDs create complex challenges to bridging the treatment gap in mental health care. A pathway was formed between general medical practitioners and a system of treatment providers who received ongoing training, feedback and support. This approach achieved completion rates and outcomes commensurate with clinical trials of ED treatments. A key finding was the benefit of a care coordinator to connect users to services and navigate barriers to ongoing treatment.

## Supplementary Information

**Additional file 1: Figure S1**. Flow of Participants Through Study. **Table S1**. Pre- and Post-Treatment Remission Status. **Table S2.** Baseline Characteristics of Service Users (N = 221 commencing treatment).** Table S3**. Targeted Micro-skills training for Practitioners.

## Data Availability

Data is owned by and subject to third party restrictions. Data may be available with permission of the Australian Government Department of Health; please contact authors with reasonable requests.
