# Supplementary material for: Evaluating an implementation model of evidence-based therapy for eating disorders in non-specialist regional mental health settings
Source: J Eat Disord. 2022 Nov 17;10:170. doi: 10.1186/s40337-022-00695-7 (PMC9670061; doi:10.1186/s40337-022-00695-7)
Supplement: Supplementary file 1 — Additional file 1: Figure S1. Flow of Participants Through Study. Table S1. Pre- and Post-Treatment Remission Status. Table S2. Baseline Characteristics of Service Users (N = 221 commencing treatment). Table S3. Targeted Micro-skills training for Practitioners. [file 40337_2022_695_MOESM1_ESM.docx]

*Additional Figure A1.* Flow of Participants Through Study

**Referrals**

**n = 303**

**Ineligible for trial ( n = 10)**

**Accessed alternative treatment pathway (n = 78)**

**Commenced Treatment**

**n = 215**

**Analysed**

**n = 143**

**Ongoing Treatment ( n = 34)**

**No outcome data (n = 38)**

- No BMI reported (n = 14)
- No ED-15 reported (n = 14)
- Anomalous data (e.g., impossible weight fluctuations; n = 10)

Additional Table A1. *Pre- and Post-Treatment Remission Status*

|  | Completers *N* (%) | | | Intention-to-treat sample *N* (%) | | |
| --- | --- | --- | --- | --- | --- | --- |
|  | Whole sample  *N* = 103 | Baseline BMI < 18.5  *N* = 22 | Baseline BMI ≥ 18.5  *N* =81 | Whole sample  *N* = 143 | Baseline BMI < 18.5  *N* = 31 | Baseline BMI ≥ 18.5  *N* = 112 |
| Baseline |  |  |  |  |  |  |
| No ED behaviours | 27 (26.2%) | 8 (36.4%) | 19 (23.5%) | 37 (25.9%) | 11 (35.5%) | 26 (23.2%) |
| ED cognitions score in normal range | 45 (43.7%) | 11 (50%) | 34 (42.0%) | 55 (38.5%) | 13 (41.9%) | 42 (37.5%) |
| Both ED criteria normative | 19 (18.4%) | 6 (27.3%) | 13 (16.0%) | 25 (17.5%) | 7 (22.6%) | 18 (16.1%) |
| Post intervention |  |  |  |  |  |  |
| No ED behaviours | 70 (68.0%) * | 17 (77.3%) * | 53 (65.4%) * | 89 (62.2%) * | 21 (67.7%) * | 68 (60.7%) * |
| ED cognitions score in normal range | 82 (79.6%) * | 19 (86.4%) * | 63 (77.8%) * | 100 (69.9%) * | 24 (77.4%) * | 76 (67.9%) * |
| Both ED criteria normative | 63 (61.2%) * | 16 (72.7%) * | 47 (58.0%) * | 78 (54.5%) * | 20 (64.5%) * | 58 (51.8%) * |
|  |  |  |  |  |  |  |
| Moved to normative (ED cognitions, behaviour) post intervention | 44 (42.7%) | 10 (45.5%) | 34 (42.0%) | 53 (37.1%) | 13 (41.9%) | 40 (35.7%) |
| Plus achieved normative BMI ≥ 18.5 | - | 8 (36.4%) | - | - | 9 (29.0%) | - |

Additional Table A2: *Baseline Characteristics of Service Users (N = 221 commencing treatment)*

| Characteristic | Mean *(SD)* | Range |
| --- | --- | --- |
| Age | 26.5 *(13.6)* | 14.0 - 67.7  *< 18 years 36.4%* |
| Duration of Eating Disorder | 5.4 *(7.1)* years | 0-31 years |
|  | *N* | % |
| Female | 133 | 93.0% |
| Occupation |  |  |
| Unemployed | 21 | 14.7% |
| Employed | 39 | 27.3% |
| Student | 66 | 46.2% |
| Retired | 5 | 3.5% |
| Not specified | 16 | 11.2% |
| Socioeconomic Status^1^ |  |  |
| Within 1 *SD* of Australian mean | 215 | 100% |
| In lowest quartile | 19 | 8.8% |
| DSM-5 Diagnosis |  |  |
| Anorexia Nervosa | 27 | 18.9% |
| ARFID | 2 | 1.4% |
| Binge Eating Disorder | 21 | 14.7% |
| Bulimia Nervosa | 32 | 22.4% |
| OSFED | 53 | 37.1% |
| Insufficient Clinical Information | 8 | 5.6% |
| Previous treatment for ED | 43 | 30.1% |
| Presence of comorbidity at baseline^2^ |  |  |
| Anxiety | 109 | 76.2% |
| Depression | 75 | 52.4% |
| Stress | 15 | 10.5% |
| PTSD | 11 | 7.7% |
| OCD | 4 | 2.8% |
| Bipolar disorder | 5 | 3.5% |
| Borderline Personality Disorder | 3 | 2.1% |
| Bullying | 4 | 2.8% |
| Substance misuse | 2 | 1.4% |
| Other not specified | 17 | 11.9% |
| Any comorbidity | 127 | 88.8% |

Notes: ^1^SES = SEIFA: Socioeconomic Index for Areas (Australian Bureau of Statistics, 2016) where Australian mean is 1000 (*SD* = 100); SES data available for *N* = 215 of 221 cases; ^2^more than one comorbidity could be selected*.*

Additional Table A3: *Targeted Micro-skills training for Practitioners*

| **Format** | - Live; 20-minutes of content and 10-minutes of question time - Recorded for viewing by those unable to attend - Copies of the presentation supplied - One page of “reflective learning” questions considering how to apply to own practice |
| --- | --- |
| **Content** | **Session 1: Making an informed diagnosis**   - Know what tools are available for making a diagnosis. - Understand how to make a provisional diagnosis from self-report questionnaires. - Understand how diagnosis impacts on choices made about treatment. |
|  | **Session 2:** **Setting up non-negotiables of therapy**   - Understand what a therapy non-negotiable is - Understand the purpose of non-negotiables and how the benefit the client - Understand how to set up non-negotiables |
|  | **Session 3:** **Using collaborative open weighing or blind weighing techniques**   - Understand what collaborative open/blind weighing is - Understand the rationale and purpose of weighing - Develop an understand of how to do these types of weighing |
